# Prevalence and Risk Factors Associated with Musculoskeletal Discomfort in Spay and Neuter Veterinarians

**DOI:** 10.3390/ani3010085

**Published:** 2013-02-04

**Authors:** Sara C. White

**Affiliations:** Spay ASAP Inc., Hartland, VT 05048, USA; E-Mail: swhitevt@mac.com; Tel.: +1-802-356-6783

**Keywords:** spay, neuter, veterinarian, ergonomics, pain, MSD

## Abstract

**Simple Summary:**

This study examined musculoskeletal pain and discomfort in spay and neuter veterinarians using an internet-based questionnaire. Hand pain was most common in the right thumb and wrist, and body pain was most common in the lower back, shoulders, and neck. Several work-related risk factors for discomfort were discovered, including long career in spay and neuter, increasing weekly hours in surgery, and decreasing job satisfaction. Although most respondents felt posture during surgery was important, few spay and neuter veterinarians have received any instruction in posture or ergonomics in surgery.

**Abstract:**

A cross-sectional study to investigate musculoskeletal discomfort (MSD) surveyed 219 veterinarians who currently or previously perform spays and neuters at least 4 hours per week. Participants were asked about the presence and severity of hand and body MSD during the previous month, whether MSD interfered with work or daily activities, whether they attributed their MSD to their spay/neuter work, and whether MSD had ever necessitated absence from work. The period prevalence of MSD was 99.1%, with 76.7% experiencing hand or wrist pain and 98.2% experiencing body pain. Hand discomfort was most commonly reported in the right thumb and/or thumb base (49.8%) and the right wrist (37.9%). Body discomfort was most commonly reported in the lower back (76.7%), shoulders (72.6%), and neck (71.7%). Increasing career length, increasing weekly hours in surgery and decreasing job satisfaction were the work-related factors with the greatest relative contribution accounting for variation in hand pain severity and total pain. Although 94.4% of respondents felt that posture during surgery is important, only 30.6% had received any instruction in posture and positioning for surgery. Future interventions should aim to optimize surgical efficiency, surgeon work schedules, and working environment. Analysis and intervention studies are required to determine further causes of MSD in these veterinarians and develop interventions to prevent MSD.

## 1. Introduction

During the past four decades, efforts to reduce the euthanasia of unwanted dogs and cats have led to the development of high-volume spay-neuter clinics [1]. These spay-neuter clinics are designed to provide spay-neuter services to targeted populations of animals on a regular and ongoing basis [2]. Since the opening of the first spay-neuter clinic in Los Angeles in 1969 [1], hundreds of spay-neuter clinics and programs have opened in the US [3], and many animal shelters provide spay-neuter services in-house for animals within their care [2]. The publication of the Association of Shelter Veterinarians Veterinary Medical Care Guidelines for Spay-Neuter Programs [2] has established this new field of high volume spay-neuter as a viable practice area within veterinary medicine. 

There is anecdotal evidence that spay-neuter veterinarians commonly experience musculoskeletal discomfort (MSD), but until now, this has not been studied. While several studies have surveyed veterinarians or human surgeons to determine the prevalence and risk factors associated with MSD, none have focused specifically on veterinary surgeons.

Scuffham *et al.* [4] has provided a review of the existing research on musculoskeletal pain and injury in veterinarians. The most thorough studies of veterinary MSD prevalence and risk factors include surveys of veterinarians practicing in New Zealand [4] and Australia [5] using modified Nordic musculoskeletal questionnaires. Scuffham *et al.* [4] studied the prevalence of musculoskeletal discomfort in New Zealand veterinarians and determined work-related and psychosocial risk factors for MSD. The overall one-year period prevalence of MSD in the surveyed veterinarians was 96%, with lower back (73%), shoulders (59%), neck (58%) and the wrists/hands (52%) most commonly affected. Over half of the respondents reported MSD in four or more body sites. Several procedures, including surgical procedures of less than 1 hour, were associated with increased risk of MSD. In addition, psychosocial factors including dissatisfaction with the level and difficulty of work, the inability to vary the pace of work, dissatisfaction with work organization, and a poor organizational culture were all associated with higher severity of MSD. 

A survey of veterinarians in Queensland, Australia [5] found a similar distribution of MSD to that in the New Zealand veterinarians [4], but with slightly lower rates of MSD. The lower back region had the highest MSD prevalence (63%), with other commonly affected areas including the neck (57%), shoulder (52%), upper back (34%), and hands/wrists (32%). This study also examined the association of MSD with psychosocial factors, and found positive associations between MSD and stressors such as career structure and time pressure. Neither study attempted to distinguish work-related from non-work-related MSD.

In human healthcare, several studies using modified Nordic questionnaires have been undertaken to quantify the MSD experienced by physicians in general [6,7], and surgeons specifically [8]. In a study of physicians in mainland China [6], the overall MSD prevalence was 67.5%, with the most common symptoms reported in the lower back (43.7%), followed by the neck (42.3%), shoulder (37.8%) and upper back (29.0%). Female physicians were more than twice as likely as their male counterparts to experience MSD in any region. A study of general surgeons in Hong Kong [8] found a high prevalence rate of work-related MSD symptoms, most commonly in the neck (82.9%), low back (68.1%), shoulder (57.8%) and upper back (52.6%). Performing open (versus laparoscopic) surgery was associated with increased low back pain. In a survey of surgeons in Germany, 95% of the surgeons stood while operating, and 84% considered their working posture to be uncomfortable or painful. Pain prevalence was highest in the back (85%), the head and neck (60%), and in the shoulders and upper arms (39%). Approximately one third of surgeons in this study used analgesic medications or had undergone physiotherapy to treat their MSD [9]. 

Surgery is a demanding job, requiring mental skills including intellectual preparation, problem solving, and emergency response capability, as well as physical skills including fine motor ability and physical endurance [10]. The work of the high volume spay-neuter surgeon demands a variety of physical tasks including standing, bending, lifting and reaching [11]. The central task of the spay-neuter surgeon is the surgery workload itself, often including more than 30 surgical procedures daily. These procedures are of limited variety compared with general surgery, and frequently involve static postures and repetitive manual tasks. Repetitiveness of work has been associated with increases in upper limb discomfort, tendinitis, and carpal tunnel syndrome in people who engage in manual work [12], and static postures, or isometric positions where little movement takes place, multiply the risk for musculoskeletal disorders attributable to those postures [13]. Thus, work in high volume spay-neuter has many qualities that would appear to put veterinarians at risk for MSD.

The primary objective of this research was to make a preliminary assessment of the prevalence and distribution of MSD in spay-neuter veterinarians in order to identify demographic, work-related, and psychosocial risk factors associated with MSD prevalence. A secondary aim was to describe self-reported postural and physical behaviors and MSD treatments employed by spay-neuter veterinarians during and outside of the surgery day.

## 2. Methods

### 2.1. Participants

Since there is no organization or registry that counts or tracks the number of veterinarians in spay-neuter practice, survey participants were recruited from several venues providing education and networking for spay-neuter and shelter veterinarians. Electronic messages containing a link to an online survey were posted on the Association of Shelter Veterinarians listserve with 538 members and the HQHVSNveterinarians (High-Quality, High-Volume Spay-Neuter Veterinarians) listserve with 147 members, inviting veterinarians to participate in the survey. Initial messages were sent on 14 August 2011, and a reminder message was sent to each listserve on 2 September 2011. In addition, electronic mail messages were sent on 22 August 2011 to the 533 attendees of The Spay Neuter Industry Professionals (SNIP) Summit conference who had provided email addresses. There was substantial overlap in membership between the groups that received surveys.

Veterinarians receiving these electronic messages were asked to participate in the survey if they currently or previously worked at least 4 hours per week performing spays and neuters for a period of at least 1 month. Recipients were further asked to forward the message to any other veterinarians meeting these inclusion criteria. Responses were collected between 14 August and 16 September 2011.

### 2.2. Questionnaire

An online survey was administered using Survey Monkey, a web-based survey service. The survey included five sections: demographics, workload, musculoskeletal discomfort, activities and positioning, and psychosocial stressors. Survey responses were anonymous and IP addresses were not collected. Participants were directed to complete the survey only once.

Demographic information collected included birth year, graduation year, number of years in spay-neuter, number of years in current or most recent spay-neuter job, gender, and whether currently working in spay-neuter. Workload information included hours per day and hours per week in surgery, hours per day at work, numbers of male and female dogs and cats altered, and other surgeries performed per day, week, and year. 

The musculoskeletal discomfort questionnaire was based on the Cornell Musculoskeletal Discomfort Questionnaire, a modified version of the Standard Nordic Questionnaire for musculoskeletal discomfort, and the Cornell Hand Discomfort Questionnaire [14,15]. The questions about aches, pains, and discomfort assessed musculoskeletal pain in 11 different body regions (neck, shoulders, upper arms, elbows, forearms, upper back, lower back, hips/thighs/buttocks, knees, feet, and ankles) as well as headache. The questions about hand pain, discomfort, or numbness assessed discomfort in the wrist and in six regions of the hands, thumb, and fingers for the right and left hand. Anatomical diagrams of hands were used to designate hand regions. Participants were asked whether they were right or left handed, or, if ambidextrous, in which hand they hold their needle holders. For all body parts, participants were asked how often they had experienced discomfort in that body part during the past month of spay-neuter, how uncomfortable the region was (slightly, moderately, or very), and whether the discomfort interfered with normal daily activities (not at all, slightly interfered, or substantially interfered). Participants were also asked whether they believed that their discomfort in each region was due to their work (yes, no, maybe or partly due to work) and whether they had ever, during their entire career in spay-neuter, missed work due to discomfort in each region.

In order to determine positions, behaviors and devices used during the surgery day and the frequency of their use, participants were asked how often they use the following devices or do the following activities: anti-fatigue floor mat; sitting for surgery; standing for surgery; shoes chosen for cushion or support; orthotic shoe inserts; wear any type of brace, splint, or other supports (other than shoe inserts); adjust table height so that elbows and wrists are approximately level; adjust table to other preferred height; listen to music; and try to maintain good posture. Respondents were also asked about outside activities that they judged to be helpful in maintaining their comfort during surgery, and physical or postural therapies and medical interventions that they had undertaken. In addition, participants were asked about their opinions about the importance of posture while performing surgery, and whether they had received training in posture for surgery.

Questions evaluating psychosocial risk factors were developed from the modified job content questionnaire [16] administered by Scuffham *et al*. [4]. Participants were asked to respond to each of 21 questions on a 3-point Likert-type scale (satisfied, neutral, not satisfied), or to respond that the question was not applicable to their work. Questions fell into six psychosocial categories: (1) contact and cooperation with management; (2) level of difficulty of work; (3) opportunity to vary pace of work; (4) work organization; (5) number of rest breaks taken; and (6) organizational culture. Participants were also asked to rate the overall level of stress they experienced in their spay-neuter job (not at all stressful, mildly stressful, moderately stressful, very stressful, or extremely stressful).

### 2.3. Data Management and Analysis

Surgical workload was calculated by assigning units based upon estimates of the actual amount of surgical work for each surgery type. Units were assigned so that cat spays and dog neuters equaled one surgery unit, dog spays equaled 2 surgery units, and cat neuters equaled 0.2 surgery unit. All other surgeries were set to equal 1 unit. Surgical speed for each participant was calculated by dividing the number of daily surgery units by the number of hours per day in surgery. Overall job satisfaction was calculated by converting the 3-point Likert scale to a numerical scale, and calculating the mean score for all answers for each participant.

Pain severity for each body region was calculated for each participant based upon the scoring guidelines accompanying the Cornell Musculoskeletal Discomfort Questionnaire [14]. The Cornell scoring guidelines were modified to reflect the one-month recall period of the current study, versus the one-week recall period of the unmodified Cornell questionnaires. Frequency scores were assigned: never = 0; 1–4 times a month = 1.5; a few times a week = 3.5; daily = 5; several times a day = 10. Discomfort scores were assigned: slightly uncomfortable = 1; moderately uncomfortable = 2; very uncomfortable = 3. Daily interference scores were assigned: not at all = 1; slightly interfered = 2; substantially interfered = 3. Pain severity was obtained by multiplying the frequency, discomfort, and interference scores for each body part. Total body pain severity for an individual was obtained by summing all the body pain severity scores for that individual. Total hand pain scores were obtained by summing the hand pain severity scores for that individual. Total overall pain scores were obtained by summing the hand pain and body pain scores for that participant. Data analysis was performed using the statistical computing program R, version 2.1.5.1. [17].

### 2.4. Univariate Analysis

Three suites of univariate analysis were performed: Fisher’s Exact Tests on categorical data; logistic regression for binary response variables (e.g., presence of pain); and simple linear regression for continuous response variables (e.g., severity of pain). The primary purpose of the univariate analysis was to guide the development of candidate multivariate models (Section 2.5). As a result, no correction was made for potential bias arising from multiple comparisons; we were looking for broad patterns. Prior to analysis, all variables were assessed for their adherence to central assumptions for a given test; non-conforming variables were transformed accordingly. Due to the prevalence of participants reporting at least some body pain (*i.e.*, limited negative responses), the response variables of the logistic regressions were limited to the presence or absence of any hand pain or of any head pain. Logistic regressions were performed for the following explanatory variables: age, length of spay-neuter career, job satisfaction, stress, dog surgeries per day/week, cat surgeries per day/week, surgery units per day/week, and surgery speed (units/hour). For the simple linear regressions, several response variables were assessed: the number of hand areas with pain, overall hand pain severity, the number of body areas with pain, overall body pain severity excluding hands, and total pain severity. The same explanatory variables were used in the linear regressions as in the logistic regression analyses, with the addition of surgery hours per day/week.

### 2.5. Multivariate Analysis

An information theoretic approach was employed to examine the multivariate relationships among the potential explanatory variables [18,19]. Candidate models were developed based on the results of the univariate tests and sample size considerations (e.g., not all participants provided answers to all questions). Candidate models were developed for three response variables (any hand pain: multiple logistic regression, n = 218; total hand pain: multiple linear regression, zero values removed, n = 162; total pain: multiple linear regression, zero values removed, n = 216). Five explanatory variables were included: CAREER (length of career in S/N), SATISFACTION (job satisfaction), TIME (total hours in surgery per week), LOAD (total surgery units per week), and SPEED (surgery units per hour). Within each response variable’s test, models are ranked by their relative likelihood or inferential potential using the Akaike Information Criterion (AIC). Any model with a ΔAIC less than 2 can be considered to have strong support, any model with a ΔAIC between 2 and 7 should be considered informative, and models with a ΔAIC > 7 likely are not very useful for subsequent inference. In addition, each model has a weight that is proportional to the relative likelihood of the candidate model. All weights for models containing a given explanatory variable can be summed to provide an index to a variable’s overall importance.

## 3. Results and Discussion

### 3.1. Demographics and Work Characteristics

A total of 219 surveys were completed. Respondents included 196 (89.5%) females and 22 (10%) males, and one participant who did not disclose gender (0.5%). Of the participants, 204 (93.2%) were currently working in spay-neuter, and 15 (6.8%) were no longer working in spay-neuter. The median age of participants was 41 years, with a range of 26 to 76 years of age. Median time since graduation from veterinary school was 11 years, with a range of 0 to 46 years. Participants had worked in spay-neuter for a median of 4.0 years (range 1 month to 28 years), and had worked in their current or most recent spay-neuter job for a median of 2.58 years (range 1 month to 28 years).

Participants spent a median of 21 hours a week in surgery, with a range from 4 hours to over 35 hours weekly. They performed a median of 91 surgeries each week, ranging from 14 to 260 weekly surgical procedures. The mean number of weekly cat surgeries was 55 (range 0 to 175) and the mean number of weekly dog surgeries was 38 (range 0 to 150). The median number of surgical units per week was 84, with a range of 7.2 to 269 units weekly. The median surgery speed was 4.72 units per hour, with a range of 0.65 to 12.88 units per hour. 

### 3.2. Musculoskeletal Discomfort Prevalence

**Figure 1:**
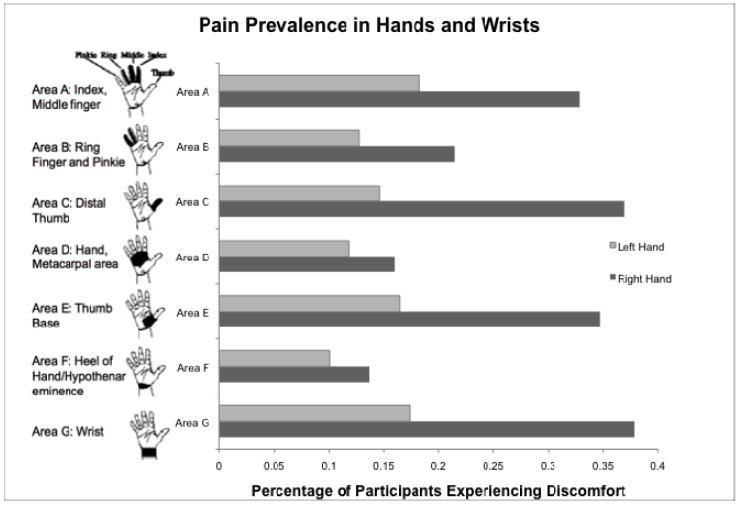
Percentage of participants experiencing MSD of any region of the hands and wrists in the past month of spay-neuter work (Hand images used with permission of the Human Factors and Ergonomics Laboratory at Cornell University).

**Figure 2:**
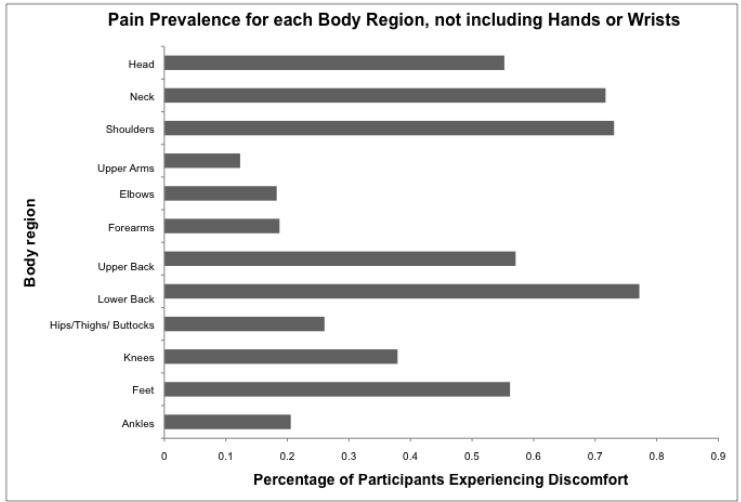
Percentage of participants experiencing MSD of any region of the body in the past month of spay-neuter work.

The self-reported period prevalence of MSD was 99.1%, with 217 of 219 participants reporting pain. Of these, 168 (76.7%) reported hand pain, 215 (98.2%) reported body pain, and 121 (55.3%) reported headaches. The reported pain affected daily activities for 168 (67.6%) of participants. In the hands, the most commonly reported areas of MSD were the right wrist (37.9%), the right distal thumb (first proximal and distal phalangeal area; 37%), and the right thumb base (first metacarpal area; 34.7%) (Figure 1). MSD was reported in some portion of the right thumb [phalangeal and metacarpal areas] by 49.8% of participants. Body MSD was most commonly reported in the lower back (76.7%), shoulders (72.6%), and neck (71.7%) (Figure 2). 

Participants were most likely to attribute thumb (81.7%), hand (74.5%), and finger (75%) discomfort completely to their work in spay and neuter. Over 50% of participants experiencing wrist, forearm, elbow, shoulder and upper back discomfort attributed their discomfort completely to their work. Overall, respondents attributed 91% of reported instances of MSD entirely or in part to spay-neuter work.

The 99% one-month period prevalence of MSD in this study is comparable to the one-year period prevalence found in a recent survey of New Zealand veterinarians [4] and is higher than that reported in Australian veterinarians [5]. The body areas with greatest MSD prevalence were the same in the current study as in these previous surveys, with the low back having the greatest MSD prevalence in all studies. In the current survey, however, the prevalence of neck, shoulder, and hand/wrist MSD was substantially higher than reported in previous studies. This difference may have to do with the specific postural and ergonomic stressors encountered by spay-neuter surgeons compared to veterinarians in general practice. 

MSD is a common finding in workplace and population surveys [4,5,6,7,8,9,10,20]. Musculoskeletal loads during work, including static postures, awkward postures, repetition, and force, are positively correlated with MSD in the body regions subjected to the loads [21]. Static body postures are common during open surgery, and there is increased postural stress from the forward bending of the neck. [8,13]. A previous study that measured neck and back muscle activity in surgeons using electromyography (EMG) found that performing open surgery was associated with significantly higher muscle activities in the cervical erector spinae and upper trapezius muscles compared with endovascular and laparoscopic procedures [22]. This increased muscle activity was associated with a significant increase in musculoskeletal pain in the postoperative versus the preoperative period in the open surgeons but not in the endovascular or laparoscopic surgeons. 

There were only 13 (5.9%) left-handed respondents, and both the right-handed and left-handed surgeons experienced greater mean number of areas of pain and greater mean pain score in the right hand than in the left. In the US population, left-handedness is estimated at about 10% of the population, although approximately one third of these individuals are “mixed-handed”, preferring to do some tasks with the right hand and some with the left. Females in the US are less likely to be left-handed than males, with about 6–7% of US females concordantly left handed [23]. In the present study, the 5.9% left-handed prevalence is lower than that reported in the US population, and likely represents the lower prevalence of left-handedness in females, and the possible tendency of mixed-handed individuals to perform right-handed surgery due to the prevalence of right-handed surgery instruments.

Previous reports of MSD in veterinarians [4,5] and human surgeons [8] have not included MSD questions about specific hand regions. Surgery is largely a manual task, and many spay-neuter surgeons anecdotally report specific hand, digit, or wrist pain. By including the Cornell Hand Discomfort Survey questions, this study was able to localize and quantify this discomfort. In the future, this information may prove to be important for the teaching of ergonomic surgery techniques and perhaps for the development of surgical instruments.

Males and females did not differ in their tendency to suffer hand (Fisher’s Exact Test: P = 0.18) or head pain (P = 0.15) or to have missed work due to any pain (P = 0.18). In other, population-wide surveys of MSD, researchers have found that females report a higher prevalence and greater severity of MSD than males [24,25]. Similarly, a higher prevalence of MSD in any body region was found for female physicians in China [6]. However, studies of MSD in veterinarians have either not studied gender differences in pain experienced by participants [4,5], or have noted a greater risk in male veterinarians for work-related chronic MSD, severe injuries, and dog and cat bites and scratches [26]. In the current study, the small number of male respondents (10% of sample) may have contributed to the lack of differences in MSD reported by male and female veterinarians. This gender distribution is similar to that of veterinarians responding to other surveys of shelter veterinarians [27,28] of 11 to 16% and is likely representative of the demographics of veterinarians in the field of spay-neuter.

Despite the high prevalence of MSD in survey participants, only 35 (16%) had ever been absent from their work in spay-neuter due to MSD. Previous studies have used absence from work as a proxy for the severity of MSD [4] instead of asking participants to rate the severity of their pain. However, the results of the current study show that many veterinarians continue to work despite experiencing moderate to severe pain. In many instances, veterinarians experiencing MSD may feel unable to miss work, aware that their work may be left undone in their absence. Relief veterinarians may not be available, or may be unable to fulfill the duties of the spay-neuter veterinarian unless already experienced in high-volume spay-neuter. In order to avoid interference from these factors in the rating of MSD severity, the current study used participant ratings of pain intensity, frequency, and effect on daily activities as a means of creating pain severity scores.

### 3.3. Psychosocial Factors

Participants were most likely to report that they were “satisfied” with the times of day they were expected to work (75.4%, 159/211), the level of enjoyment of the work (74.3%, 159/214), the total number of working hours per week (71.1%, 150/211), the level of difficulty of the work (69.8%, 150/215), cooperation among the participant and co-workers (70.4%, 150/213), and work as a whole (69.5%, 148/213). Participants were most likely to report being “not satisfied” with the way their organization is run (37.9%, 81/214) and the number of rest breaks (33.5%, 71/212).

Thirty-three participants (15.3%) rated their job in spay neuter as “very” or “extremely” stressful, whereas 97 participants (44.9%) rated their job as “mildly” or “not at all” stressful. The remaining 86 participants (39.8%) consider their job “moderately” stressful.

### 3.4. Risk Factors for MSD

The univariate analyses produced only two logistic relationships were statistically significant: age and head pain (β = −0.06, SEβ = 0.02, P = 0.0003, OR = 0.95), and career length and head pain (β = −0.07, SEβ = 0.03, P = 0.01, OR = 0.93). Younger veterinarians and those with a shorter career in spay-neuter were more likely to experience headaches. This fits well with studies of headache prevalence that indicate that the occurrence of both migraine and non-migraine headaches peaks in between ages 30 and 39 [29,30]. In addition, in adults, headaches of all types are more common in women than in men [24], and migraines are approximately 3 times more common in adult premenopausal women than in men of the same age [29]. In the current study, as in the rest of veterinary medicine, females are more highly represented in younger age groups. Males represented 12.2% of veterinarians in this sample aged 40 and over, but just 7.4% of the veterinarians under 40. This gender disparity may have accounted for some of the increased headache prevalence in the younger veterinarians. The relationship between age and career length is likely responsible for the relationship between career length and headache. Headache prevalence was not related to any of the other workplace or psychosocial factors studied, and the addition of headache pain to the analysis did not alter the overall discomfort prevalence reported in the current study.

Of the explanatory variables assessed using univariate linear regression, only career length, job satisfaction, stress, and surgery hours per week had consistent effects on the extent of pain (Table 1). Of the response variables, the number of hand areas experiencing pain appeared to be the most responsive to the working environment. 

**Table 1:** Univariate linear regression results (all statistically significant relationships have positive slopes).

	Hand Pain		Body Pain		Overall
Factor	# of areas	Severity	# of areas	Severity	Severity
*Demographic*					
Age	F = 0.27, P = 0.60	**F = 5.06, P = 0.03**	F = 0.86, P = 0.35	F = 0.91, P = 0.34	F = 4.00, P = 0.05
Career in S/N	**F = 5.57, P = 0.02**	**F = 21.16, P < 0.001**	F = 0.61, P = 0.43	**F = 4.51, P = 0.04**	**F = 17.68, P < 0.001**
*Psychosocial*					
Job Satisfaction	**F = 0.05, P = 0.003**	**F = 4.93, P = 0.03**	**F = 6.43, P = 0.01**	F = 3.09, P = 0.08	**F = 6.09, P = 0.01**
Stress	**F = 13.05, P < 0.001**	**F = 6.42, P = 0.01**	**F = 7.56. P = 0.006**	**F = 8.76, P = 0.003**	**F = 11.83, P < 0.001**
*Practical*					
Dogs/day	**F = 4.95, P = 0.03**	F = 0.30, P = 0.59	F = 1.22, P = 0.27	F = 2.13, P = 0.15	F = 1.46, P = 0.23
Cats/day	F = 1.25, P = 0.27	F = 0.34, P = 0.56	F = 0.22, P = 0.64	F = 1.07, P = 0.30	F = 0.97, P = 0.33
Units/day	**F = 5.64, P = 0.02**	F = 0.68, P = 0.41	F = 1.45, P = 0.23	F = 3.34, P = 0.07	F = 2.57, P = 0.11
Dogs/week	**F = 6.66, P = 0.01**	F = 0.72, P = 0.39	F = 1.33, P = 0.25	F = 1.60, P = 0.21	F = 1.67, P = 20
Cats/week	**F = 5.82, P = 0.02**	F = 3.93, P = 0.05	F = 1.07, P = 0.30	F = 2.08, P = 0.15	**F = 4.51, P = 0.04**
Units/week	**F = 8.48, P = 0.04**	F = 2.15, P = 0.14	F = 1.88, P = 0.17	F = 2.42, P = 0.12	F = 3.46, P = 0.06
Speed (units/hr)	F = 2.12, P = 0.15	F = 0.005, P = 0.94	F = 1.25, P = 0.26	F = 1.33, P = 0.25	F = 0.52, P = 0.47
Sx hours/day	F = 1.42, P = 0.23	F = 0.89, P = 0.35	F = 0.01, P = 0.91	F = 0.49, P = 0.49	F = 1.01, P = 0.31
Sx hours/week	**F = 10.28, P = 0.002**	F = 3.92, P = 0.05	**F = 4.52, P = 0.03**	F = 2.64, P = 0.11	**F = 4.98, P = 0.03**

The results of the univariate analysis were used to develop candidate models for multivariate analysis. The three response variables that were tested were any hand pain, total hand pain, and total pain, and the explanatory variables included CAREER (length of time in S/N), SATISFACTION, TIME (total surgery time per week), LOAD (total units per week), SPEED (units/hour).

Any Hand Pain, Eleven candidate models had a ΔAIC < 2, suggesting strong relative support (Table 2). The low model weights suggest that none of models offer much explanatory power. Based on weight sums, the relative importance of the explanatory variables is CAREER (Σω = 0.53) > SPEED (Σω = 0.52) > TIME (Σω = 0.51) > SATISFACTION (Σω = 0.49) > LOAD (Σω = 0.47).

**Table 2:** Any hand pain results (k = number of parameters).

	k	ΔAIC	Weight (ω)
Career	1	0.0000	0.0368
Career + Speed	2	0.1000	0.0366
Career + Time + Speed	3	1.0400	0.0349
Career +Load + Speed	3	1.0400	0.0349
Career + Time	2	1.1200	0.0348
Time	1	1.2800	0.0345
Time + Speed	2	1.5000	0.0341
Speed	1	1.6200	0.0339
Load + Speed	2	1.7300	0.0337
Career + Satisfaction	2	1.9500	0.0334
Career + Load	2	1.9700	0.0333
Career + Time + Load	3	2.0300	0.0332
Career + Satisfaction + Speed	3	2.0800	0.0331
Time + Load	2	2.3500	0.0327
Career + Time + Load + Speed	4	2.9400	0.0317
Career + Satisfaction + Load + Speed	4	2.9500	0.0317
Career + Satisfaction + Time + Speed	4	2.9900	0.0317
Load	1	3.0300	0.0316
Satisfaction	1	3.0400	0.0316
Career + Satisfaction + Time	3	3.0400	0.0316
Satisfaction + Time	2	3.2200	0.0313
Time + Load + Speed	3	3.4200	0.0310
Satisfaction + Time + Speed	3	3.4700	0.0309
Satisfaction + Speed	2	3.6200	0.0307
Satisfaction + Load + Speed	3	3.6500	0.0306
Career + Satisfaction + Load	3	3.9300	0.0302
Career + Satisfaction + Time + Load	4	4.0100	0.0301
Satisfaction +Time + Load	3	4.3300	0.0296
Global	5	4.8700	0.0288
Satisfaction + Load	2	5.0000	0.0286
Satisfaction + Time + Load + Speed	4	5.3700	0.0281

Total Hand Pain, Five candidate models had a ΔAIC < 2, suggesting strong relative support (Table 3). The low model weights suggest that none of models offer much explanatory power. Based on weight sums, the relative importance of the explanatory variables is TIME (Σω = 0.60) > CAREER (Σω = 0.57) > SATISFACTION (Σω = 0.52) > LOAD (Σω = 0.51) > SPEED (Σω = 0.50). 

**Table 3:** Total hand pain results (k = number of parameters).

	k	ΔAIC	Weight (ω)
Career + Satisfaction + Time + Load	4	0.0000	0.0445
Career + Satisfaction + Time	3	0.7203	0.0430
Career +Time + Load	3	0.9069	0.0426
Career + Satisfaction + Time + Speed	4	1.3812	0.0416
Global	5	1.8542	0.0406
Career + Time	2	2.3254	0.0396
Career +Time + Load + Speed	4	2.6180	0.0391
Career + Time + Speed	3	2.8054	0.0387
Satisfaction + Time + Load	3	3.7790	0.0369
Time + Load	2	4.2483	0.0360
Satisfaction + Time	2	4.6189	0.0354
Satisfaction + Time + Speed	3	5.3012	0.0342
Satisfaction + Time + Load + Speed	4	5.5752	0.0337
Time	1	5.7424	0.0334
Time + Load + Speed	3	5.8976	0.0332
Career + Satisfaction	2	6.0012	0.0330
Time + Speed	2	6.2566	0.0326
Career	1	6.7489	0.0318
Career + Satisfaction + Load + Speed	4	7.0404	0.0313
Career + Satisfaction + Speed	3	7.3815	0.0308
Career + Satisfaction + Load	3	7.7311	0.0303
Career + Speed	2	7.9864	0.0299
Career + Load + Speed	3	8.3751	0.0293
Career + Load	2	8.6724	0.0289
Satisfaction + Load + Speed	3	13.2950	0.0229
Satisfaction	1	13.9383	0.0222
Load + Speed	2	14.1369	0.0220
Satisfaction + Load	2	14.9930	0.0210
Speed	1	15.3710	0.0207
Load	1	15.3816	0.0206
Satisfaction + Speed	2	15.4846	0.0205

Total Pain, Three candidate models had a ΔAIC < 2, suggesting strong relative support (Table 4). The low model weights suggest that none of models offer much explanatory power. Based on weight sums, the relative importance of the explanatory variables is TIME (Σω = 0.57) > CAREER (Σω = 0.56) > SATISFACTION (Σω = 0.55) > LOAD (Σω = 0.52) > SPEED (Σω = 0.50).

**Table 4:** Total pain results (k = number of parameters).

	k	ΔAIC	Weight (ω)
Career + Satisfaction +Time	3	0.0000	0.0437
Career + Satisfaction + Time + Speed	4	0.8085	0.0420
Career + Satisfaction + Time +Load	4	1.4731	0.0406
Career + Satisfaction + Load	3	2.3131	0.0390
Global	5	2.6961	0.0382
Satisfaction + Time	2	3.0435	0.0376
Career + Time	2	3.3571	0.0370
Satisfaction + Time + Speed	3	3.6311	0.0365
Career + Satisfaction + Load + Speed	4	4.1144	0.0356
Career + Time + Speed	3	4.3919	0.0351
Satisfaction + Time + Load	3	4.4190	0.0351
Career + Time + Load	3	5.1900	0.0337
Satisfaction + Time + Load + Speed	4	5.4973	0.0332
Career + Time + Load + Speed	4	5.8471	0.0327
Career + Satisfaction	2	6.0098	0.0324
Time	1	6.1407	0.0322
Career + Load	2	6.2319	0.0320
Career + Satisfaction +Speed	3	6.3050	0.0319
Satisfaction + Load	2	6.7198	0.0313
Time + Speed	2	6.9748	0.0309
Time + Load	2	7.9117	0.0295
Career + Load + Speed	3	8.1615	0.0291
Satisfaction + Load + Speed	3	8.2985	0.0289
Career	1	8.3668	0.0288
Time + Load + Speed	3	8.3998	0.0287
Career + Speed	2	8.9420	0.0280
Load	1	10.4245	0.0260
Load + Speed	2	12.2050	0.0238
Satisfaction + Speed	2	12.8095	0.0231
Satisfaction	1	13.0429	0.0228
Speed	1	14.9289	0.0207

The use of an information theoretic approach in this context allows for the ranking of candidate models by their relative explanatory power; high ranking models (*i.e.*, ΔAIC < 2) explain more variation in the response variable (*i.e.*, have stronger relative support) than the other tested candidate models. The weight of a given model is indicative of the amount of variation explained by a particular model. A model can provide strong support relative to other candidate models while not providing much absolute explanatory power. The results of these analyses suggest that the assessed explanatory variables (e.g., CAREER) are contributors to MSD but that there other factors at play that were either not assessed in the questionnaire or were not assessed in a way that allowed for the development of models with more explanatory power.

There was a consistent relationship between the amount of time spent working in spay-neuter and the severity of hand and overall MSD experienced. An increase in MSD severity was found both for increasing years working in spay-neuter practice, and for increasing hours per week spent spaying and neutering. While one might expect that increases in age could explain part of the increase in MSD for those with increasing career length, the univariate analysis found that age was positively related only with hand pain severity, whereas increases in spay-neuter career length was positively related to increases in number of areas of hand pain, severity of hand pain, severity of body pain, and severity of overall pain. Thus, age explains only a portion of the increased MSD attributable to career length in spay-neuter.

Similarly, the increase in MSD experienced by those working more weekly surgical hours was not because they were performing a higher numbers of surgeries. Increased surgical units per week (regardless of number of hours worked) only resulted in an increase in the number of areas of hand pain, with no effect on number of areas of body pain or overall pain severity. However, increasing hours in surgery per week was positively related to increases in number of areas of hand pain, number of areas of body pain, and overall pain severity. Thus, it appears that the weekly hours in surgery, rather than the number of surgeries performed, was the greater factor in MSD prevalence and severity.

While increasing *weekly* hours in surgery is related to greater MSD prevalence and severity, the number of *daily* hours in surgery is not related to reported MSD. Surgeons working fewer, longer days have the same MSD risk as surgeons working the same number of weekly surgery hours spread over several days.

Surgical speed appears to have little relationship to MSD. Surgeons completing only a few surgical units per hour had similar MSD prevalence and severity to surgeons completing two or three times as many surgical units per hour. Two possibilities might explain this. First, the ergonomic strain of the sustained static posture used for surgery may be similar for a given duration, regardless of the number of procedures performed. Second, surgical speed may have less to do with the speed of the operator’s hands, and have more to do with the efficiency of surgical techniques used and the efficiency of the operating room. The speedier surgeon may not be performing faster movements, but instead may be completing surgeries with fewer movements, and also waiting through less “down time” between procedures while in surgery. 

The interplay between weekly hours in surgery, surgical speed, and MSD suggests workplace and work schedule modifications that could mitigate the risk of MSD for spay-neuter veterinarians. High weekly surgical hours is one of the easiest workplace factors to modify. An obvious way of decreasing surgical hours would be simply to implement schedules with fewer surgical hours per surgeon. However, increases in workplace efficiency may allow surgeons to complete the same amount of work in less time without sacrificing quality or income. This would be expected to decrease the risk of MSD since, unlike work hours, speed and total surgery numbers are not related to MSD. One way to increase efficiency is to decrease the surgeon’s amount of idle time between surgeries. This may be achieved by increasing the staff-to-veterinarian ratio so that all non-veterinary tasks are performed by veterinary technicians and assistants, and by increasing equipment such as surgery tables and anesthesia machines; this way, the surgeon does not have to wait for patient transport between surgeries but can simply don new sterile gloves and proceed to the next surgery. This could be accomplished without sacrificing potentially beneficial “micropauses” in workflow. Pauses of as little as 15–30 seconds executed multiple times per hour, especially if combined with stretches or exercises, may be beneficial in reducing MSD [31]. 

A second way to increase efficiency in spay-neuter surgery is to implement surgical techniques that are themselves more efficient. Instruction in high volume surgery techniques is available via continuing education wet labs and on DVD’s and downloadable videos [32]. These techniques, taught by boarded veterinary surgeons, are minimally invasive and safe for patients, and generally decrease the surgeon’s time to complete each surgery. Further benefits of these techniques include decreased patient time under anesthesia, a decreased risk of patient cooling due to decreased surgery time and decreased body cavity exposure, and decreased tissue trauma.

In addition to implementing as many measures to increase efficiency as possible, it may be wise to place some limitations on the surgical hours expected of each surgeon, in order to preserve the health and career longevity of spay-neuter surgeons. For some, full time work may need to include some non-surgical time in order to avoid the MSD risks associated with increasingly high hours per week in surgery. 

Low job satisfaction and high job stress were also positively related to increases in MSD prevalence and severity. This relationship between psychosocial factors and MSD has been noted in many other studies [4,5,25]. Increases in feelings of stress may lead to muscle tension and an increase in MSD, or conversely, increases in work-related MSD may make work more stressful and less satisfying. It is likely that both of these are true to some extent and in some participants, in other words, that psychosocial stressors can be both the cause and the result of MSD. 

### 3.5. Posture, Activities, and Treatments

Respondents reported using a variety of positions and devices during the surgery day. Most participants (184 responses, 84%) reported standing during surgery “always” or “most of the time,” whereas only 17 (7.8%) surgeons usually sit for surgery. The remaining respondents alternate between sitting and standing during the surgery day. 

Most participants reported using an anti-fatigue floor mat (164 responses “always” or “most of the time”; 74.8%) and shoes chosen for comfort and support (185 “always” or “most of the time”; 84.5%), and some respondents used orthotic shoe inserts (36, 16.4%). Standing surgeons may experience decreased fatigue and discomfort in the back and lower limbs with the use of a floor mat, particularly if the duration of standing is at least 3–4 hours [33,34,35]. The mats associated with the least fatigue and discomfort during prolonged standing tend to be those characterized by increased elasticity, increased stiffness, and decreased energy absorption [33]. Cushioned shoes [34] and insoles [35] also provide increased comfort, and a combination of cushioned footwear and floor mat provides the best results. 

Most surgeons preferred to adjust the surgery table so that their elbows and wrists remain level most of the time (162 responses “always” or “most of the time”; 74%). Adjusting the height of the surgical table to suit the patient size and the force required during the surgery may reduce positional ergonomic stressors. A table that is too low may lead to an excessively forward bent back and neck, whereas a table that is too high may result in elevated shoulders and abducted upper arms. Optimal table height should allow for relaxed shoulder and upper arm positions and minimize bending of the spine. Surgeries requiring greater application of force, such as adult dog castrations, may be easiest to perform with a slightly lower table height to enhance leverage [36]. Positioning the patient closer to the surgeon may also alleviate ergonomic stress on the neck, shoulders, and back [13]. In some cases, alleviating postural stress on one body region will result in increased postural stress in a different region [37]. The surgeon may be best served by adopting a variety of positions throughout the day, by alternating between seated and standing surgeries, or by selecting positions that decrease postural stress on problem areas. 

For surgeons who sit, the use of a saddle-shaped seat instead of a standard surgical stool may allow the surgeon to maintain a more neutral lower back position [37] and may allow the patient to be positioned nearer to the surgeon while avoiding raised shoulder or arm positions, even during surgeries of large, deep-bodied patients. However, some people experience increases in leg discomfort when using saddle chairs [38] so individual preference and comfort should be considered.

The repetitive hand and wrist motions required during surgeries put these body regions at risk. Awkward grips, twisting motions, and application of force with a bent or deviated wrist will exacerbate the ergonomic risks to the hands and wrists [21]. Surgeries on larger patients may require the use of greater force. Physical compression, such as the chronic pressure of the surgical instruments on the digits, can be an additional risk factor for hand MSD [36]. These risks can be mitigated somewhat by improving surgical technique in order to eliminate unnecessary use of awkward or bent hand positions. Surgical training should emphasize proper instrument-handling techniques and hand positions, and surgeons could benefit from re-evaluating their own technique periodically to ensure that they are not placing unnecessary stress on their hands and wrists. Videotaping may assist the solo surgeon in analyzing and correcting their instrument-handling techniques. 

Additional factors that may affect the forces placed on surgeons’ hands include surgical instrument selection and maintenance and suture needle sharpness. Some needleholders and hemostatic forceps may require several kilograms of force to engage the ratchet, and a lateral push of over 1 kilogram to disengage the ratchet [39]. This force may be repeated hundreds of times a day, each time the surgeon opens or closes an instrument. Selecting instruments appropriate to the surgeon’s hand size and to the surgical task may allow for selection of instruments requiring less force. Regular instrument lubrication and maintenance may also decrease the amount of force required to operate the instruments. Ensuring that suture needles are sharp will also decrease the amount of force that must be applied while suturing, and the gentler tissue handling that results may be beneficial for the patient as well as the surgeon [39].

Many respondents reported that their comfort during the surgery day was improved by physical activities outside of the workday, most commonly sports and aerobic activities (95 responses; 43.4%), stretching (93 responses; 42.5%), strength or weight training (69 responses; 31.5%), and yoga (50 responses; 22.8%). A physically active lifestyle has been associated with lower prevalence of MSDs, although it is not clear whether physical activity prevents MSD, or whether those with less MSD are more active [40,41]. Once MSDs are present, exercise therapy can result in improvement of chronic discomfort, and staying active after lower back injury can reduce the duration of sick leave [42].

Seventy-nine (36.1%) respondents had used a physical or alternative therapy to ease their discomfort, including massage (55 responses; 25.1%), chiropractic (29 responses; 13.2%), physical therapy (16 responses; 7.3%), acupuncture (7 responses; 3.2%), and one response (0.5%) each for occupational therapy and Alexander Technique. Twenty-three respondents used more than one of these therapies. Manual therapies such as chiropractic and massage can be effective at relieving acute and chronic low back pain, neck pain, certain extremity joint conditions, and some types of headache [43]. An intensive, multidisciplinary approach that incorporates occupational and clinical therapies has been shown to produce greater improvements in pain and function for chronic low back pain compared to non-multidisciplinary rehabilitation [42].

One hundred thirty-six participants (62.1%) reported using NSAIDs to maintain their comfort during surgery. The study did not distinguish between regular versus intermittent use of NSAIDs. In addition, 15 (16.8%) of these NSAID users also used a prescription medication for pain, and 7 (3.2%) used muscle relaxants. All users of prescription pain medications and muscle relaxants also used NSAIDs. The rate of use of NSAIDs in the current study, while apparently high, is actually typical for residents of Western countries, where up to 70% of the adult population uses over-the-counter analgesics regularly [44]. NSAID use has been shown to decrease pain and improve function in patients with chronic low back pain [42]. Muscle relaxants can also be effective at reducing pain, particularly in the case of acute MSD [42]. Other treatments for pain include using heat or cold application (49 responses; 22.4%) and receiving injections for pain (4 responses, 1.8%). Fourteen participants (6.4%) have had surgery for a painful condition that they believe was caused by, or was exacerbated by, their work in spay-neuter surgery.

Twenty-nine participants (13.2%) wear some type of brace or splint at least sometimes while not performing surgery, while seven participants (3.2%) use braces, splints or supports during surgery. Questions did not distinguish types of brace or splint, or the body regions being supported, or the frequency or duration of use. In some cases, a splint or brace may be effective for supporting an injured limb and providing pain relief through immobilization. Many health care providers recommend wrist splinting at night for symptoms of carpal tunnel syndrome. While evidence for the benefit of this practice is inconclusive, studies have also noted few if any negative effects [45]. Back belts worn during work have not been shown to be effective at preventing low back pain [46], and lumbar supports are ineffective as treatment for low back pain [42]. Prolonged use of lumbar supports may result in decreased muscular strength in the trunk, as well as a false sense of security [42]. 

Many surgeons responded that they “try to maintain good posture” during the surgery day. Open-ended responses for how participants attempted to maintain good posture include postural answers (e.g., stand up straight; try not to slouch or hunch over; stay skeletally aligned or balanced), behavioral answers (e.g., cuing postural self-awareness and self adjustment to surgical events such as adjusting posture at the beginning of each surgery) and patient and equipment-positioning answers (e.g., position table at a specific height, use a platform or stool to change their height relative to a fixed-height or inadequately adjustable table). In addition, many surgeons had between-surgery tension-relief routines including specific stretches, yoga, or dancing. Many listened to music during surgery (141 responses “always” or “most of the time”; 64.4%), which may also encourage body movement and tension relief. 

While 26% of respondents were satisfied with their posture during surgery, another 50% of participants admitted being unsure what constitutes “good” posture for surgery. About one third of respondents (36%) responded that they did not know how to make changes in their posture in surgery, and 25% of respondents answered that it was too hard to change their postural habits. Half of respondents felt that they start the day with good posture but then fall into old habits, and 42% get too tired or fatigued during the surgery day to maintain good posture. Twenty-nine percent responded that they needed to concentrate on the patient during surgery, not their posture. Only 3.2% of respondents answered that they believed posture was unimportant during surgery, while 94.4% agree or strongly agree that posture in surgery is important.

Most spay-neuter veterinarians have received no instruction of any type in posture or ergonomics during surgery, although a few have received instruction from multiple sources. Only 27 participants (12.3%) received instruction during veterinary school, and one (0.4%) received instruction during internship or residency. Twenty six participants (11.9%) received postural or ergonomic instruction during veterinary continuing education, and 21 (9.6%) have had instruction in non-veterinary venues, such as from a healthcare provider. Thirty-six participants (16.4%) have studied posture and ergonomics independently; for 23 of these 36 participants, this independent study was their only postural or ergonomic instruction. A total of 152 (69.4%) participants have had either no formal instruction in posture and ergonomics, or have learned about posture and ergonomics via independent study only. Indeed, training in ergonomics and posture is uncommon in the training of human surgeons as well [47,48], and ergonomics in surgery and surgeons’ postural health are just beginning to receive attention by researchers [13,47,49]. 

Postural and ergonomic training interventions have mixed results for prevention of MSD in workplaces. For manual handling tasks, *i.e.*, those that require force to lift, lower, push, pull, carry, move, hold or restrain a person, animal or object, training interventions have generally been ineffective at preventing MSD [50]. For computer users, training alone can result in transient improvements in posture and positioning, while postural training combined with ongoing videography or photo feedback can result in a more sustained improvement in posture [51]. There has been some success for postural intervention in surgeons. A prospective study of a postural training and awareness intervention (Alexander Technique) in laparoscopic surgeons resulted in improved surgical ergonomics, speed, and dexterity, as well as subjective improvements in posture during surgery [47]. 

The optimal intervention strategy for prevention of and recovery from work-related MSD for spay-neuter veterinarians remains unknown. For the spay-neuter surgeon, the most useful strategy may be a combination of methodologies that include education, postural training, visual feedback, and workplace and work schedule modifications. Education could include information about ergonomic risk factors, equipment choices, patient positioning, and how to select and adjust equipment in order to minimize postural risks. Training in appropriate positioning and use of hands and body could ideally take place during initial surgical technique training, when surgeons are forming habits, or might occur later in the surgical career. Video or pictoral feedback could enhance learning of appropriate postures and would allow for ongoing monitoring of body positions. Workplace and work schedule modifications would have to occur on a case-by-case basis. A participatory ergonomics approach would likely be useful, in that it would allow individuals to use their own knowledge to control and enhance their working conditions [52].

In the veterinary field, such interventions would be useful if made available both to practicing spay-neuter veterinarians, as well as to veterinarians newly entering the field. More broadly, since other studies have shown a high rate of MSD in general practice veterinarians as well [4,5], veterinary students and general practitioners may also benefit from training in the use of ergonomics and appropriate posture in surgery and in other areas of general practice.

### 3.6. Methodological Considerations

A one-month period prevalence was chosen for this study for several reasons. Recall periods of greater than two months are likely to underestimate injuries [53]. The self-reported annual incidence rate for at-work injuries may be over 30% greater when a one-month recall period is used instead of a 12 month period [54]. Conversely, short recall periods of less than 3 months have been shown to have good correlation with weekly reports of MSD [55]. The standard recall period for the Cornell Musculoskeletal Discomfort Questionnaire is 1 week; however, with this survey’s low minimum requirement for weekly hours in spay-neuter work (4 hours/week), and the possibility for some intermittent pain, a one-month recall period was chosen. An additional reason for using a one-month recall period versus a one-year recall period is to allow inclusion of as many participants as possible. Since spay-neuter is a relatively new field in veterinary medicine, many veterinarians currently working in spay-neuter have worked in this field for less than one year. Indeed, 30 participants, or 13.7%, had worked in spay-neuter for one year or less at the time of the study.

Much of the MSD reported in the current study could not be explained by the demographic, work-related, and psychosocial factors included in the analysis. The low model weights in the multivariate analysis signify low explanatory power, indicating that the factors analyzed do not explain most of the variability in the prevalence and severity of MSD in spay-neuter veterinarians. Other, non-workplace factors are likely to explain the variability in pain experienced. Activity level, physical fitness, genetics, smoking, alcohol use, and history of past pain or injury may all be factors that contribute to MSD, but these were not included in this study.

Similarly, additional non-surgery-related workplace factors that may be associated with MSD were not evaluated in the present study. In the veterinary setting, lifting heavy animals is a risk factor for injury [56]. In many high volume surgical practices, veterinary technicians and assistants are primarily responsible for moving and lifting animals, but in some cases veterinarians may perform or assist in lifting tasks. Additional workplace tasks including computer work and motor vehicle use [57] may also increase the risk of MSD.

A further limitation of this study is that, due to the cross-sectional design, it is not possible to discern cause and effect. It is possible that some spay-neuter veterinarians are now working less, or are slower surgeons, because of pain they have experienced. Also, it is possible that some veterinarians who were experiencing the most pain or job stress have already left the field and no longer participate in conferences or listservs related to the field, so were not aware of the study. The exclusion of these workers no longer in the field may have caused a decrease in reported adverse health effects, known as the “healthy worker effect” [58]. Conversely, there could be a response bias such that the people experiencing more MSD may be more likely to complete a survey concerning MSD, thus resulting in an increase in reported MSD. 

## 4. Conclusions

While not a recognized veterinary specialty, high volume spay-neuter surgery requires unique skills that require practice and that are enhanced by specialized training. Replacing skilled spay-neuter surgeons can be difficult for employers [11], so staff retention becomes essential to the productivity and effectiveness of high volume spay-neuter practices. Efforts should be made to mitigate or minimize risk factors for MSD in working spay-neuter veterinarians in order to retain these veterinarians in the field. Postural and ergonomic interventions and training, as well as making improvements in efficiency, speed, and work scheduling, may be useful in maintaining surgeon comfort and retaining workers in the spay-neuter field. 

The present study showed that spay-neuter surgeons are at high risk for experiencing MSD (99.1%) that they attribute entirely or in part to their work (91%) and that affects their daily activities (67.6%). The results of multivariate analyses suggest that increasing career length, increasing weekly hours in surgery, and decreasing job satisfaction were the most important work-related factors contributing to hand pain severity and overall pain severity. Future interventions should aim to optimize surgical efficiency, surgeon work schedules, and working environment. Analysis and intervention studies are required to determine further causes of MSD in these veterinarians and develop interventions to prevent MSD.
